# Pediatric integrative medicine: pediatrics' newest subspecialty?

**DOI:** 10.1186/1471-2431-12-123

**Published:** 2012-08-15

**Authors:** Sunita Vohra, Soleil Surette, Deepika Mittra, Lawrence D Rosen, Paula Gardiner, Kathi J Kemper

**Affiliations:** 1 CARE Program for Integrative Health & Healing, Department of Pediatrics, Faculty of Medicine and School of Public Health, University of Alberta, Stollery Children's Hospital, Edmonton, AB, Canada; 2 CARE Program for Integrative Health & Healing, Department of Pediatrics, Faculty of Medicine, University of Alberta, Edmonton, AB, Canada; 3 Mind Body Medicine, Edmonton, AB, Canada; 4 Whole Child Center, Oradell, NJ, USA; 5 Program for Integrative Medicine and Health Care Disparities; Department of Family Medicine, Boston University School of Medicine, Boston, MA, USA; 6 Wake Forest University Health Sciences, Winston-Salem, NC, USA; 7 CARE Program, Department of Pediatrics, Stollery Children's Hospital, University of Alberta, 8B19 Edmonton General Hospital, 11111 Jasper Avenue, T5K 0L4, Edmonton, Alberta, Canada

## Abstract

**Background:**

Integrative medicine is defined as relationship-centered care that focuses on the whole person, is informed by evidence, and makes use of all appropriate therapeutic approaches, healthcare professionals and disciplines to achieve optimal health and healing, including evidence-based complementary and alternative medicine. Pediatric integrative medicine (PIM) develops and promotes this approach within the field of pediatrics. We conducted a survey to identify and describe PIM programs within academic children’s hospitals across North America. Key barriers and opportunities were identified for the growth and development of academic PIM initiatives in the US and Canada.

**Methods:**

Academic PIM programs were identified by email and eligible for inclusion if they had each of educational, clinical, and research activities. Program directors were interviewed by telephone regarding their clinical, research, educational, and operational aspects.

**Results:**

Sixteen programs were included. Most (75%) programs provided both inpatient and outpatient services. Seven programs operated with less than 1 FTE clinical personnel. Credentialing of complementary and alternative medicine (CAM) providers varied substantially across the programs and between inpatient and outpatient services. Almost all (94%) programs offered educational opportunities for residents in pediatrics and/or family medicine. One fifth (20%) of the educational programs were mandatory for medical students. Research was conducted in a range of topics, but half of the programs reported lack of research funding and/or time. Thirty-one percent of the programs relied on fee-for-service income.

**Conclusions:**

Pediatric integrative medicine is emerging as a new subspecialty to better help address 21st century patient concerns.

## Background

The National Institutes of Health (NIH) defines complementary and alternative medicine (CAM) as a group of diverse medical and health care systems, practices, and products that are not generally considered part of conventional medicine [1]. Integrative medicine is defined as relationship-centered care that focuses on the whole person, is informed by evidence, and makes use of all appropriate therapeutic approaches, healthcare professionals and disciplines to achieve optimal health and healing [2], and as such includes the best of evidence-based CAM therapies and evidenced-based conventional therapies.

Integrative medicine is similar to the biopsychosocial model of medicine [3] in that it focuses on the whole person, but it also articulates a commitment to evidence-based practice using multiple therapeutic modalities, including CAM therapies.

Rates of reported CAM usage among children vary between studies, but prevalence is notable across populations. Approximately 10-40% of healthy children and more than 50% of children with chronic, recurrent, or incurable conditions use CAM, most often in conjunction with conventional care [4,5]. Although many families use CAM along with conventional care, only 20% to 65% discuss their CAM use with their physician, non-reporting usually occurs because they do not think it is relevant [6-10]. This communication gap may adversely affect patient safety related to interactions between CAM and conventional care.

The hesitance that families have in discussing their health related preferences, values, and beliefs raises significant concern for pediatricians providing family-centered care. Integrative pediatrics is meant to address this concern by equipping clinicians with the education to address families’ health related preferences and communication about CAM. Most pediatricians have stated they are interested in learning more about CAM therapies, and are starting to feel more comfortable referring to CAM providers [11].

By 1998, over 60% of US medical schools had incorporated some education about CAM into their curriculum [12]. Founded in 1999, the Consortium of Academic Health Centers for Integrative Medicine (Consortium) has grown in membership from 8 to 48 accredited North American medical schools that engage in research, education, and clinical initiatives in integrative medicine. Integrative clinical services are typically offered in one clinic or specialty (such as chronic pain services, oncology, women’s health or family medicine), rather than throughout academic health centers [13,14].

Paralleling the increased visibility of integrative medicine in academic health centers, academic pediatric integrative medicine (PIM) programs are also developing and promoting an evidence-based integrative approach within children's hospitals. Few studies have evaluated the state of pediatric integrative medicine.

We conducted a survey to identify and describe PIM programs within academic children’s hospitals across North America by interviewing program directors to assess their activities with respect to clinical, research, and educational initiatives in pediatric integrative medicine. Key barriers and opportunities were identified for the growth and development of academic PIM initiatives in the US and Canada.

## Methods

A survey was developed by content experts, pilot tested, revised, and sent by email to 20 North American programs (the survey is available upon request). We used a snowball technique for sampling, i.e. respondents were asked to identify additional academic PIM programs until no more programs were identified by any respondent. Inclusion criteria were having clinical, research, and education activities; programs meeting only one or two of these criteria were noted, but did not meet inclusion for this study. The survey was deployed on multiple occasions within an 18 month period, until no additional programs were identified by any of the previous respondents.

For each included program, interviews were conducted with directors of the included PIM programs between March and August 2007. Each interview was conducted by two study authors (DM and SV or PG) and lasted 60–120 minutes. Information was collected on clinical, research, educational, and operational aspects of the programs in order to capture information on the achievements, challenges and opportunities that were faced, and advice was sought on the factors critical to success or failure, including staffing and resources required for program initiatives (the interview guide is available from study authors upon request). Descriptive analyses were conducted (means, frequencies). Since program directors were encouraged to forward the survey invitation to other programs, non-response could not be calculated. Ethics approval was granted for this study from the University of Alberta Health Research Ethics Board.

## Results

**Figure 1:**
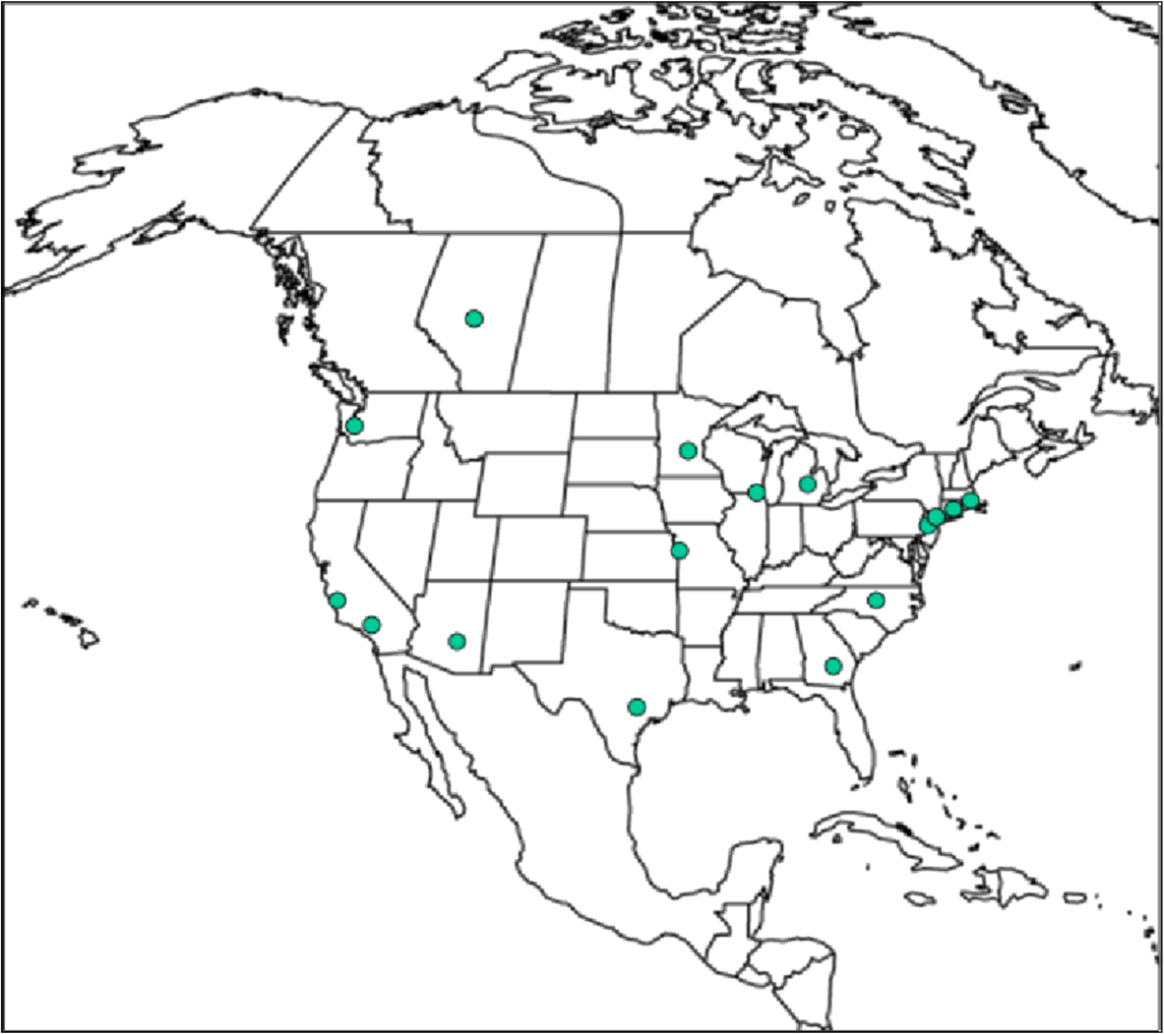
Location of included academic pediatric integrative medicine programs.

Of the 143 accredited medical schools in North America in 2007, 16 met our inclusion criteria as having an academic PIM program (see Figure 1). The reasons for starting a PIM program were varied. One hospital wished to be a leader in the field, while several programs were initiated in response to philanthropic interest. Programs experienced a variety of support from upper administrative and hospital management, ranging from “benign neglect” to “very supportive”. Two of the programs were the second ones to be initiated by a given individual: of these, one closed after the “champion” left, but the other remains active. The first program was initiated in 1991, the most recent, at the time of data collection, in 2007.

### Clinical services

All programs provided clinical services, and 75% had both an outpatient and inpatient service. The most common conditions addressed by the inpatient services were cancer (64%), chronic pain (50%), and gastrointestinal or other chronic illness (28.5% each). The most commonly addressed outpatient conditions were cancer (57%), mental health (50%), and chronic pain or abdominal pain or headaches (42.8% each). Abdominal pain, mental health issues, and headaches were more commonly addressed in outpatient (42.9%, 50%, 42.9%) than inpatient services (14.3%, 0%, 7%). Two of the programs dealt solely with pain. Most programs served all children (0–16 or 0–21). One outpatient program did not see children under 3 and another saw primarily adolescents. Two programs continued to see chronically ill patients after they had become adults.

Referral experiences varied between centers. For example, self-referrals were accepted by 86% of outpatient vs. 57% of inpatient services. In one case, pediatric referral was required in order to create dialogue with physicians around CAM and to insure that patient load could be accommodated. Physician referral was preferred in the inpatient settings. One service only accepted physician referral because they did not want to be perceived as going behind any physician’s back, while another accepted self-referral if the patient’s insurance accepted it.

The gatekeeper model of having one person make care decisions predominates in both the inpatient and outpatient settings. Only one program had a team approach to inpatient care, while four programs had this for outpatient care.

The range of CAM services offered in inpatient or outpatient settings varied (see Table 1). The most comprehensive inpatient oncology service provided acupuncture, acupressure, massage therapy, reflexology, aromatherapy, Reiki, herbal counseling, nutrition and yoga to all interested patients without contraindications. CAM practitioners who provided inpatient services included: massage therapists, acupuncturists, and a naturopath, a music therapist, an art therapist, and a yoga instructor. However, most inpatient services offered CAM therapies through conventional health care providers, who were not always licensed in the CAM modality that they provided. Some outpatient clinics offered on-site CAM services, but often patients were referred to CAM practitioners in the community who had been vetted is some manner. Acupuncture/acupressure, mind-body and energy therapy were the most commonly offered modalities in both inpatient and outpatient services. Energy therapy was available in 64% of inpatient programs but only 29% of the outpatient services. Further details are available in Table 2.

**Table 1:** Complementary and alternative therapies offered by pediatric integrative medicine programs in North America

**Academic therapy**	**Creative arts (music, art)**	**Nutritional counseling**
Acupressure/Acupuncture	Exercise physiology	Osteopathy/CST
Aromatherapy	Homeopathy	Reiki/Energy healing
Biofeedback/hypnosis/mind-body medicine	Massage/Infant massage	TCM
Botanical counseling	Meditation	Therapeutic/Healing touch
Craniosacral therapy	Naturopathy	Yoga

**Table 2:** Most common complementary and alternative therapies offered on site at 16 Pediatric Integrative Medicine programs*

**Modality**	**Inpatient% (n)**	**Outpatient% (n)**
Acupuncture/Acupressure	7	6
Mind Body	6	7
Energy Therapy	9	4
Massage Therapy	7	4
Botanicals/Herbals	4	6
Nutritional Counseling	2	4
Craniosacral Therapy	0	4

*Excludes modalities offered by only 1–3 sites.

Since their inception, the 16 programs had identified a total of only three adverse events: (i) a patient wanted only massage therapy but energy work (e.g. reiki) was also provided; (ii) minor bruising from acupuncture; and (iii) one patient had a post-traumatic stress flashback post hypnotherapy.

#### Personnel, policies and credentials

Nine programs operated with between 1 and 11 FTE divided among MDs, RNs, psychologists, nutritionists and CAM providers. Seven programs had less than 1.0 FTE clinical personnel, usually an MD.

There was a wide variability in methods for credentialing both conventional and complementary practitioners to provide CAM. Credentialing of acupuncturists, massage therapists and other CAM professionals varied substantially across the programs and between inpatient and outpatient services. Sometimes credentialing was an internal hospital process; other times it was external, for example, through state licensure. In one inpatient program the CAM provider came in under the auspices of a family “visitor”, but this meant that they could not chart their visit. In another case, an inpatient program with a physician provider of CAM was put on hold for six months in order for the physician to obtain the proper CAM credentials. Outpatient programs often referred patients to community providers vetted by the program’s physician or through word of mouth.

Programs stated the importance of making sure that the CAM providers were comfortable working within the conventional system and had some pediatric experience or formal training with children, and that the modalities chosen reflected the community’s interests.

Most of the programs had few, if any, policies and procedures in place at their onset, and a few lacked formal administrative support at the time of the interviews. Only 31% of programs reported having a policy on natural health products (NHP)/dietary supplements (DS), and these varied in what products were addressed. Existing natural health products policies included guidelines for pharmacy approval of patients’ herbal supplements as well as dietary supplement policies for outpatient/inpatient programs. Many of the programs indicated an interest in developing/obtaining institutional policies for CAM and NHPs. Clinical and administrative challenges were identified as barriers.

### Educational initiatives

The 16 programs offered a variety of elective educational initiatives ranging from clinical electives for medical students and residents to community outreach presentations. Almost all (94%) described programs for residents in pediatrics and/or family medicine (25% of which were mandatory), 81% reported educational programs for medical students (19% of which were mandatory), 56% had training opportunities for fellows, and one program has a dedicated pediatric integrative medicine fellowship program. More than half (56%) offered continuing education opportunities for faculty and/or community physicians. One third (33%) provided programs for nurse and nurse practitioners, and six offered some education or training for families.

Only two programs offered research training: one mentored research projects, the other offered research-specific training in pediatric integrative medicine.

Educational strategies included: lectures, presentations (local, international, stakeholders, health professionals, general public), rounds, conferences, lunch & learns, information sheets, training at CAM schools, online educational resources, newspaper and magazine articles, and TV interviews. One quarter of PIM programs offered some online training.

Team members involved in PIM education consisted at a minimum of pediatricians, nurses, and medical students (undergraduate, graduate, and postgraduate). The inclusion of CAM practitioners was variable.

Most (81%) of the programs were interested in a collaborative pediatric training program, but identified funding and time as barriers. Two frequent comments were that: i) interest would increase with the presence of funding or ii) interest existed, but programs were already overwhelmed by current commitments.

### Research

Half of the programs had become inactive in research due to lack funding and/or time and resources. Research initiatives most commonly comprised health services research (50%) and randomized controlled trials (31%). Clinical research topics included massage therapy, antioxidants and music therapy for cancer patients; and guided imagery, hypnotherapy, Reiki, and acupuncture for chronic pain. Dietary supplements were also a frequent topic of research. No PIM program reported conducting basic research on CAM, though basic research may have been conducted in other departments in academic health centers.

Research funding was mostly obtained through peer-reviewed grants, foundations and philanthropy. Amounts ranged from tens of thousands to several million dollars. At the time of the interviews, no research funding had been obtained from industry.

Research was published in both conventional and CAM journals. Some programs emphasized the need to publish in mainstream medical journals in order to avoid “preaching to the choir” and to further educate the broader medical community on pediatric CAM.

#### Operations

Most PIM programs were located in an affiliated hospital or medical school and had some salaried employees. Space was a concern and/or a limiting factor for several programs. Funding came from varied sources: philanthropy, research grants, institutional, and tuition. Almost one third (31%) relied on fee-for-service income. Inpatient costs were often covered through integration into existing programs. Costs of outpatient programs were covered in a variety of ways: some by philanthropy, some modalities were covered by some insurance, some care was billed as a physician consult, and many fees were charged out-of-pocket on a sliding scale. Programs engaged in limited promotional activities; relying mostly on websites, presentations, and brochures. Two programs did no promotion: one because they could not handle more patients, the second because the administration asked them not to due to limited space and personnel. Two programs mentioned being supported by their institution’s marketing department. Only half of the programs reported engaging in strategic planning; those that did used regular business meetings, retreats, discussions with other subspecialties and developed 1–5 year plans with which to move forward.

#### Advice for starting pediatric integrative medicine initiatives

The most common advice offered for others considering developing an academic PIM program was to build slowly and to work within the conventional system: “utilize people already within the system and call upon them to be part of the team,” “go where you are invited.” This encompassed maintaining a strong professional reputation, not alienating potential allies by being adversarial: “no turf battles,” “avoid fights you don’t have to fight,” and basing decisions on sound evidence. It was also noted that having a champion, both within the program and within the other groups that the program dealt with (e.g., administration) was very important: “leadership vision – to be in a house that wants you.” Financial considerations were essential, and issues ranged from sustainability to the importance of looking for funding in non-traditional places, such as philanthropy. It was considered difficult to maintain the programs, particularly clinical endeavors, without additional outside support. One general piece of advice was to “Lay down some policies, guidelines, program structure before program launch.” Finally, establishing rapport through sound research was very important: “research is key – helps to gain acceptance.”

## Discussion

Academic PIM is a new and growing field in North America with an already rich history, (Table 3: A Selected History of Integrative Pediatrics). CAM use is common among pediatric populations, and over 80% of pediatricians want additional information about CAM.[11] In 2005, an Institute of Medicine (IOM) report recommended that sufficient information about CAM be incorporated into undergraduate, graduate and postgraduate curriculums to enable a licensed professional to competently advise patients about CAM [15]. Academic health centers and children’s hospitals can play an important role in providing this education to pediatricians, and, as documented, some have begun this process [16].

**Table 3:** A Select History of Pediatric Integrative Medicine

	
1981	Pendergrass TW, Davis S. Knowledge and use of “alternative” cancer therapies in children. *Am J Pediatr Hematol Oncol***.1981;3**:339-345
1982	Zeltzer L, Lebaron S. Hypnosis and nonhypnotic techniques for reduction of pain and anxiety during painful procedures in children and adolescents with cancer. *J Pediatr*. 1982;101:1032-1035
1984	Kohen DP, Olness KN. The use of relaxation-mental imagery (self-hypnosis) in the management of 505 pediatric behavioral encounters. *J Dev Behav Pediatr*. 1984;5:21-25
1986	Field T, et al. Tactile/kinesthetic stimulation effects on preterm neonates. *Pediatrics*. 1986;77:654-658
1987	First Introductory Pediatric Hypnosis Workshops provided at annual meeting of the Society for Behavioral Pediatrics (now the Society of Developmental and Behavioral Pediatrics)
1994	Spigelblatt L, et al.The use of alternative medicine by children. *Pediatrics*. 1994; 94: 811–814. (First CAM use publication in Pediatrics)
1995	The Ambulatory Pediatric Association established a Special Interest Group in Integrative Pediatrics
1996	Kemper K. *The Holistic Pediatrician* (HarperCollins) [2^nd^ edition 2002]
1996	Kemper K. Separation or synthesis: a holistic approach to pediatrics. *Pediatr Rev*. 1996;17(8):263
1996	Kemper K. Seven Herbs Every Pediatrician Should Know. *Contemp Pediatr*.1986:79-81
1997	University of Arizona received the NCCAM pediatric center grant
1998	Boston Children’s launched the Center for Holistic Pediatric Education and Research
1998	Sikand A, Laken M. Pediatricians’ experience with and attitudes toward complementary/alternative medicine. *Arch Pediatr Adolesc Med*. 1998 152:1059-1064
1999	APA Presidential address on “Holistic Medicine = Good Pediatrics”
2000	AAP Task Force on CAM formed
2000	1st PIM conference in Arizona
2001	AAP Policy statement on children with disabilities using CAM
2004	PedCAM Research and Education (PedCAM) network was established
2004	The International Pediatric Integrative Medicine (IPIM) Network formed
2004	Integrative Pediatrics Council formed at PIM meeting in Minnesota
2005	AAP provisional Section on Complementary Holistic Integrative Medicine formed
2006	First article in *Pediatrics in Review* series on integrative pediatrics published
2008	AAP Taskforce on CAM report published and provisional section on Complementary and Integrative Medicine becomes a full section within the AAP
2009	“A Parent’s Guide to Complementary and Integrative Medicine” AAP brochure published
2009	Culbert T, Olness K. *Integrative Pediatrics* (Oxford University Press)
2010	*Mental Health, Naturally* published by AAP

Previous work has identified challenges associated with initiating, developing and maintaining integrative medicine programs, including: clinical and administrative barriers; inertia, general resistance to change; lack of familiarity with other providers; historical enmity between physicians and other provider groups; skepticism; lack of funding; perceived or actual lack of regulation of providers; heterogeneity of products and providers [16]. Many of these are also challenges described by the PIM programs in this study.

Sustainability, as it related to time and funding, emerged as a significant issue in the interviews. Half of the programs were inactive in research due to a lack funding and/or time and resources (including devoted FTE). These barriers were also noted by some of the programs actively engaged in research. Given current insurance funding which does not consistently cover CAM services, it is also difficult to maintain pediatric integrative clinical services without external support. Seeking guidance for accessing the various funding sources is a must as there are different standards for each one. The care provided in PIM clinics is typically low volume, with few procedures, but resource- intense in terms of health care provider time. The financial impact of this kind of care must be considered at the inception of such programs. Integrative medicine programs are potentially beneficial as a way of differentiating a health organization from competitors, increasing patient satisfaction [17]. attracting new philanthropy and as a driver for patient care innovation, but they need administrative and financial support. The majority of programs lacked formal credentialing processes for the provision of CAM. In many cases, this impacted the ability to have CAM providers fully participate in inpatient care, and sometimes for conventional health personnel to provide CAM. When credentialing exists, it usually included some combination of licensing, education, and experience. Credentialing of CAM providers is something that all new programs should be aware of and account for in their development phase, as there are few guidelines to follow. Policy around referrals to CAM providers or modalities is also not well defined in many programs. Establishing policies and procedures for credentialing and referrals will help smooth the startup process of establishing a PIM program and avoid some of the problems identified by our research.

Networking and creating relationships with other medical groups (e.g. primary care, nursing, etc.) was important in order to avoid turf battles, which can negatively affect the perceived legitimacy of the program within the institution and community.

Most programs offered educational opportunities to medical students and residents, but only about half offered continuing education opportunities to physicians and even less offered anything to nurses, CAM practitioners or to families. Educational outreach to other conventional health care professionals and to families is an important activity. There was also a lack of research training available to students, which means that new researchers in this field are not necessarily being trained. Research training was identified as a critical need in the IOM report [15]. Part of the goals of the National Center for Complementary and Alternative Medicine (NCCAM) is to provide awards to support the development of CAM researchers, in order to address this deficit.

Inpatient care costs were, for the most part, covered by existing programs at the various hospitals and thus available to all the patients. Outpatient care costs were sometimes covered by insurance or philanthropy but most often, the requirement for patients to co-pay out-of-pocket affected accessibility of the various CAM modalities.

Currently, academic PIM is heterogeneous. At the time of the interviews the most well-developed aspect of the 16 PIM programs was the clinical portion, while the education and research arms were uneven across the programs. In contrast, integrative family medicine developed a residency program offered at several medical schools that included a distributed learning fellowship in integrative medicine and expanded family medicine residency from 3 years to 4 years [18]. This program (Integrative Medicine Fellowship in Residency) is now open to other primary care specialties beyond family medicine. The same program also offers an Integrative Medicine in Residency online curriculum which can be customized and woven into primary care resident training but does not require the extra year of training [19]. PIM programs may be able to develop a similar approach if they collaborated together in residencies or fellowships. PIM could also learn from the obstacles and challenges encountered by integrative family medicine, including lack of time, dedicated financial support, and multiple locations [18].

## Conclusions

Pediatric integrative medicine (PIM) is one of pediatrics’ newest subspecialties. Like the development of pediatrics beginning in the mid 19^th^ century as a specialty within medicine to better address the need of children [20], PIM has emerged as a specialty within pediatrics to help address 21^st^ century concerns. Children are not small adults and need health care that addresses their needs; within CAM and integrative medicine, as in conventional medicine, this requires research and training specific to pediatrics. Furthermore, in the field of CAM, very little pediatric training is available to practitioners, which makes the development of PIM even more important. Just as the field of pediatrics took time to win recognition and credibility within medicine, PIM will also have to prove itself in order to be acknowledged within conventional domains.

## Abbreviations

PIM: Pediatric Integrative Medicine; CAM: Complementary and Alternative Medicine; NHP: Natural Health Product; IOM: Institute of Medicine.

## Competing interests

Three of the authors have launched academic pediatric integrative medicine programs.

## Authors' contributions

SV made substantial contributions to the conception and design of the study, acquisition of data, and its interpretation. SS made substantial contributions to the analysis and interpretation of the data, and drafted the article. DM made substantial contributions to the design of this study, acquisition of data, and its interpretation. LDR made substantial contributions to the conception and design of the study. PG made substantial contributions to the conception, acquisition of data, and its interpretation. KK contributed to the design of the survey and interpretation of its data. SV, DM. LR, PG and KK helped revise the draft article for important intellectual content. All authors have read and given approval of the version submitted for publication

## Author information

SV is founder and Director of the CARE Program for Integrative Health & Healing the first pediatric integrative medicine program in North America,and Professor, Department of Pediatrics, Faculty of Medicine and School of Public Health, University of Alberta, Edmonton, AB, Canada. LDR is the founder of the Whole Child Center, Oradell, NJ, USA. PG is the assistant director of the Program for Integrative Medicine and Health Care Disparities; Department of Family Medicine, Boston University School of Medicine, Boston, MA, USA. KJK is the Caryl Guth Chair for Integrative Medicine and Professor of Pediatrics, Social Science and Health Policy, and Family and Community Medicine at Wake Forest University Health Sciences, Winston-Salem, NC, USA.

## Pre-publication history

The pre-publication history for this paper can be accessed here:

http://www.biomedcentral.com/1471-2431/12/123/prepub
